# Purchasers’ deliberations on psychosocial needs within the process of allocating healthcare services for older home-dwelling persons with dementia: a qualitative study

**DOI:** 10.1186/s12913-018-3550-7

**Published:** 2018-10-01

**Authors:** Anette Hansen, Solveig Hauge, Ragnhild Hellesø, Ådel Bergland

**Affiliations:** 1 Department of Nursing Science, University of Oslo, Faculty of Medicine, Postbox 235, 3603 Kongsberg, Norway; 2 Department of Nursing and Health Sciences and Centre for Care Research, University of South-Eastern Norway, Postbox 235, 3603 Kongsberg, Norway; 3 Institute of Health and Society, Department of Nursing Science, University of Oslo, Faculty of Medicine, Postbox 1130 Blindern,, 0318, OSLO, Norway; 4 Lovisenberg Diaconal University College, Lovisenberggaten 15b, 0456 Oslo, Norway

**Keywords:** Healthcare assessment, Community healthcare services, Allocation, Purchasers, Home care, Psychosocial needs, Dementia, Alzheimer’s disease

## Abstract

**Background:**

Meeting psychosocial needs is a significant component of quality dementia care. To enable persons with dementia to live at home for as long as possible, a community healthcare service offering care where physical, social, psychological, cultural and spiritual needs are met, is recommended. A comprehensive allocation process is required to allocate individually tailored healthcare services. However, the allocation process for older home-dwelling persons with dementia, specifically for services to safeguard psychosocial needs, remains largely unexplored. Accordingly, this study aims to explore purchasers’ deliberations on psychosocial needs during the process of allocating healthcare services to older home-dwelling persons with dementia.

**Methods:**

The study had a descriptive design with a qualitative approach. The primary data source was focus group interviews with purchasers who assess and allocate healthcare services. The interview data were supplemented by a review of administrative decisions made by the purchasers. Data from the focus group interviews were analysed using a descriptive and interpretive approach. Content analysis of the administrative decisions was conducted.

**Results:**

The purchasers described the allocation process as challenging. The following four themes reflect the complexity of the allocation process: (i) an unfamiliar and unclear concept; (ii) a hierarchy of needs; (iii) an adjusting allocation process; (iv) a challenging documentation of administrative decisions.

**Conclusions:**

The purchasers viewed a comprehensive allocation process as important. However, a web of different interplaying aspects prevented the purchasers from conducting a comprehensive need-led allocation process. Insufficient assessment or allocation threatens the adequate safeguarding of the psychosocial needs of persons with dementia. Having varied and sufficient services to allocate is of great importance, but is not sufficient. Psychosocial needs must be better incorporated as a significant element throughout the entire allocation process.

**Electronic supplementary material:**

The online version of this article (10.1186/s12913-018-3550-7) contains supplementary material, which is available to authorized users.

## Background

To meet the great challenge posed by the increasing number of care-dependent persons, a restructuring of healthcare services characterized by a shift from institutional care to home care services has occurred [1]. Most of the 47 million persons worldwide living with dementia [2] are home-dwelling [3]. To enable persons with dementia (PWDs) to live at home for as long as possible, a community healthcare service offering care where physical, social, psychological, cultural, and spiritual needs are met is recommended [2].

In high-quality dementia care, meeting psychosocial needs (e.g. need for social contact, meaningful activities, maintenance of identity) is viewed as significant [4, 5]. However, the psychosocial concept is perceived as broad [6] and difficult to define [7]. In the literature, psychosocial is often described as a concept consisting of psychological and social factors that mutually affect each other [8]. However, there are several different perceptions of the concept, and reaching consensus on one common understanding that embraces the complexity of the concept seems difficult [6, 9]. The term psychosocial is used in numerous governing documents [10, 11], though often with sparse specification concerning the content of the concept [12].

PWDs stand at risk of socially isolation [13], and neuropsychiatric symptoms such as depression, anxiety, and internal and physical unrest (e.g., feelings of restlessness or distress, wandering, shouting) are common [14–16]. It may be difficult for PWDs to express or safeguard their own psychosocial needs, owing to disabilities caused by the dementia [1]. Previous research has shown that some of the most common unmet needs among home-dwelling PWDs are related to daytime activities, company, and psychological distress [17]. Comprehensive assessment and sufficient allocation of services are important to meeting PWDs’ psychosocial needs and to enabling PWDs to stay in their own homes for as long as possible.

A comprehensive assessment is advocated, considering the PWDs’ situation, resources, and needs in order to assess, not only physical, practical, medical, and safety needs but also social, psychological, cultural, and existential needs [18]. Purchasers, who assess applicants’ needs and allocate municipal healthcare services, often express a wish to conduct comprehensive assessment [19, 20]. However, assessing older persons’ mental, psychosocial, and existential needs is not always adequately emphasized [18], potentially resulting in these needs remaining unmet. Consequently, PWDs’ quality of life may be reduced, hindering their ability to stay at home. Physical needs appear to be more carefully assessed and emphasized than social needs [20, 21]. Constrained resources may undermine a comprehensive assessment [22]. Despite that legislation and guidelines underpin the importance of individual need-led assessment [23], a more supply-led assessment often occurs [24, 25].

Making decisions and allocating services to home-dwelling older persons are viewed as challenging [19]. As with the assessment, service allocation does not always occur in accordance with the recipient’s needs [26], but is often based more on economic resources and the services available [21, 25]. When allocating services, physical and medical needs are also emphasized over psychosocial needs [23, 27]. Since the dementia often makes it challenging for PWDs to meet their own psychosocial needs, services allocated solely to meet physical or medical needs may be insufficient. There is a risk of underestimating PWDs’ need for assistance if they do not have a functional disability in addition to their cognitive impairments [28]. Although day care centres, support groups, and other services have become common in many countries [29], the availability and capacity of such services are often limited [30, 31]. However, differences are seen, both across countries as well as across different regions of individual countries [32].

Despite the growing interest in recent decades in issues related to the assessment and allocation of services to older home-dwelling persons [33], knowledge of the allocation process regarding services for PWDs is sparse. For PWDs to experience good quality of life, safeguarding psychosocial health and needs are of great importance. Thus, it is important to obtain knowledge of purchasers’ considerations regarding psychosocial needs when assessing and allocating services for PWDs. To our knowledge, no study has explored purchasers’ deliberations on psychosocial needs in the process of allocating healthcare services for older home-dwelling PWDs.

## Methods

### Aim

This study aimed to explore purchasers’ deliberations on psychosocial needs during the process of allocating healthcare services to older home-dwelling PWDs.

In this study, the term “allocation process” incorporates both assessment and allocation.

### Design

This study had a descriptive design with a qualitative approach. Two different data sources were used: focus group interviews and administrative decisions.

### Setting

This study was conducted in four medium-sized municipalities, located in three different counties in Southeast Norway. All four municipalities comprised both rural and urban areas. A purchaser-provider split model is often used to provide healthcare services in large- and medium-sized Norwegian municipalities [34]. In this model, administration and provision of healthcare services are separated into two units: a purchaser unit and a provider unit [35]. Applications for healthcare services are sent to the purchaser unit. The PWD’s relatives or healthcare professionals often submit the application on the PWD’s behalf. The application might concern a specific service, such as day care centre, or might contain a more general request for assistance. Purchasers, working in the purchaser unit, assess the applicant’s individual resources and needs. Usually, assessment is performed through a conversation conducted in the PWD’s home, often with relatives present. Based on a comprehensive assessment the purchasers shall allocate services that best meet the PWD’s actual needs. An administrative decision that describes the applicant’s actual condition and the content and scope of the allocated service is formed [36]. The administrative decision also describes the professional assessment that has been undertaken and references the legal basis on which services are granted, including information on recipients’ right to appeal the decision [37]. The administrative decision is sent to the applicant and the providers of the healthcare services (e.g., home care services [HCSs] or day care centres). The providers deliver the allocated services stated in the administrative decisions. The Norwegian municipalities have implemented and adjusted the purchaser-provider split model to fit different local contexts [35], this was also the case in present study.

### Recruitment and participants

The recruitment process was conducted in two steps. First, an invitation to participate in the study was sent to healthcare services leaders of the in 13 municipalities, each with more than 30.000 citizens. A minimum population of 30.000 was chosen to ensure a sufficient number of administrative decisions. The first four municipalities to positively respond were included. In the second step, the leaders of the purchaser units were asked to recruit purchasers for focus group interviews and to provide 50 anonymized administrative decisions allocating HCSs and/or day care centres. The only inclusion criterion for the focus group interviews was that the purchasers had broad experience assessing and allocating healthcare services to older home-dwelling PWDs. This issue was important, because the purchasers assessed and allocated services to persons with different health problems, ages, and needs. All participants were women between 30 and 65 years of age (for participant characteristics, see Table 1).

**Table 1:** Characteristics of the focus group interview participants (*N* = 19)

Characteristics	n
Work experience in healthcare services	
8–10 years	3
11–20 years	4
21–30 years	9
31–40 years	3
Profession	
Registered nurse (some with post-baccalaureate education)	15
Social educator	2
Occupation therapist	1
Physiotherapist	1
In addition to health education at a baccalaureate level or higher, the purchasers had additional education in healthcare legislation	

The inclusion criteria for administrative decisions are described in Table 2.

**Table 2:** Inclusion criteria for administrative decisions

Administrative decisions allocating home care services, and/or	
Administrative decisions allocating day care at day care centres	
The person granted services had to be diagnosed with dementia or with symptoms indicating dementia	
The PWDs granted services had to be aged 70 years or older	

Administrative decisions were limited to those pertaining to persons aged 70 years or older (Table 2) because, in some Norwegian municipalities, younger PWDs are offered additional or different services, like day care centres at farms [38]. Because we know that numerous cases of dementia go undiagnosed [39], symptoms consistent with dementia had to be observed by the purchasers or the HCS providers if a formal diagnosis had not been made.

### Data collection

In this study, focus group interviews were the main source of data. Data were collected from September 2012 to June 2013.

### Focus group interviews

Semi-structured focus group interviews were conducted. The interview guide [see Additional file 1] consisted of relatively open-ended questions, allowing the participants to share and discuss their diverse viewpoints and experiences [40]. The focus group interviews were led by one of the researchers (AH); another of the researchers (SH) participated as an assistant-moderator in the first interview [40]. After each interview, the researcher (AH) prepared a brief note containing the main experiences and content that appeared important during the interview. Considerations related to the group dynamic or whether it could be beneficial to adjust the interview guide before the next interview were discussed by three of the researchers (AH, SH, ÅB). One focus group interview was conducted in each of the four municipalities. At their request, the interviews were held at the purchasers’ workplace, and lasted between 1.5 and 2 h.

### Administrative decisions

Each of the leaders of the four purchaser units were asked to provide approximately 50 anonymized administrative decisions allocating HCSs and/or day care centres. We received 268 administrative decisions, and 246 were included in the study, 184 allocated HCSs and 62 allocated day care centres. The 22 excluded administrative decisions did not meet the inclusion criteria; they granted services like nursing home care or care benefits.

### Data analysis

The data from the two sources were analysed separately.

### Focus group interviews

In the analysis, an iterative approach was taken, moving back and forth between the whole and parts of the data [41]. The focus group interviews were audio-recorded and transcribed verbatim shortly after each interview by the researcher who conducted the interviews (AH). The transcripts were read several times to ensure familiarity and to gain an overall impression [41]. To facilitate systematic organization of the data, NVivo 11 software was used [42]. Meaningful units were identified, coded, and categorized [43]. A search for similarities and differences between the categories was conducted, and overlapping categories were grouped together [41]. For example, the category “a broad concept” was grouped together with the category “challenging to define, describe and interpret”, resulting in a new category designated “a broad and challenging concept to define”. Finally, the categories were abstracted and formulated into four themes [41]. The iterative approach allowed a continuous movement in our understanding and interpretation of the data, resulting in new viewpoints and questions advancing the analysis. Collaboration among the four researchers was essential in the analysis process, both to provide a “wider analytic space” [44], ensuring a common understanding and interpretation, and to reach a consensus at each stage in the analysis process.

### Administrative decisions

The administrative decisions were analysed separately using content analysis [45, 46]. Because data from the focus group interviews were the main data source, the administrative decisions provided additional data to exemplify and complement the purchasers’ descriptions and reflections [43]. Meaningful units describing psychosocial health or needs were extracted and categorized either as social aspects or psychological aspects. An example of a unit categorized under social aspects was “need social contact”, and an example of a unit categorized under psychological aspects was “depressed”. Subsequently, four main categories were developed, based on describing, or not describing, social aspects and/or psychological aspects (Table 3). Finally, the number of administrative decisions in each of the four category was counted, and separated into two groups – allocating either HCSs or day care centres. By counting the descriptions, we were able to identify patterns in the descriptions (or lack thereof) of psychosocial needs [46]. By complementing the focus group interview data with the administrative decision data, we were able to obtain broader and more nuanced insights into the assessment and allocation of psychosocial needs than we could have using only the focus group interview data [43]. Additionally, these complementary data reinforce our findings and validate the interpretations and conclusions [47].

**Table 3:** Number of administrative decisions allocating HCSs and/or day care centres

Main categories	Administrative decisions allocating HCSs (*n* = 184)	Administrative decisions allocating day care centres (*n* = 62)	Administrative decisions in total (*N* = 246)
Social aspects or needs documented	36	57	93
Psychological aspects or needs documented	5	0	5
Both social and psychological aspects or needs documented	1	5	6
Social or psychological aspects or needs not documented	142	0	142

## Results

The findings reveal a challenging and complex allocation process. Four themes derived from the analysis of the focus group interviews and the administrative decisions are presented below.

### An unfamiliar and unclear concept

Psychosocial needs were perceived as an unfamiliar and unclear concept. To better understand and operationalize the concept, the purchasers distinguished between social needs and psychological needs. One explained: *“Psychosocial health and needs, we are not using the term psychosocial… we are not placing the social and the psychological in the same concept…”* Social needs were perceived as needs for social contact and interaction, whereas psychological needs were mainly perceived as needs related to depression, anxiety, unrest, and safety. The purchasers found it challenging to define social and psychological needs: *“The content of these needs is difficult to describe and explain”.* Interpreting and articulating psychological needs were perceived as especially challenging, because the purchasers perceived these needs as being closely connected to the PWD’s subjective experience. Additionally, psychological needs were viewed as sensitive and associated with a certain degree of taboo, which further challenged the interpretation and articulation of these needs. A careful approach had to be taken regarding psychological aspects. When the purchasers viewed psychological health and needs as taboo, this could result in omitting psychological aspects throughout the allocation process.

### A hierarchy of needs

The purchasers rated the assessment of various needs into different levels of difficulty and importance, and a hierarchy thus emerged. Assessing physical needs was described as the easiest and most straightforward to perform; one purchaser explained: *“…they are more measurable, the physical needs. Can you dress yourself? Yes / no…”.* Assessing social needs was experienced as being more challenging: *“It becomes woollier… How is your social network...? Are your social needs covered?”* Psychological needs were regarded as the most complicated and challenging to assess: *“It is difficult to capture and assess anxiety, we cannot measure it… it is less concrete to assess”.* Another elaborated: *“… they are much more blurry… there are more assumptions… How is your mental state? What is the anxiety about, what are the implications of the anxiety?”* The rating of needs into different levels of importance for assessment was described as a practice where physical needs were strongly emphasized when assessing for HCSs, whereas social needs were strongly emphasized when assessing applications for day care centres, with psychological needs receiving less focus in relation to both HCSs and day care centres. When psychological needs remained unassessed, decisions allocating services to meet these needs were not made.

### An adjusting allocation process

The purchasers described the allocation process as challenging and as a process in which several adjustments were made. Conducting a comprehensive assessment was perceived as significant because PWDs had an increased risk of being socially isolated and experiencing depression and anxiety. Nevertheless, a lack of time could hinder the assessment. One explained: *“…you don’t always have time to assess all the patients’ functions and needs. So, in relation to allocating HCSs, assessment of social and psychological health and needs is often neither conducted nor emphasized”.* In some cases, as an adjustment to the limited time, assessing applications for HCSs was even done by phone instead of in the PWD’s home. When assessments were made by phone, the observations that could normally be made in the PWD’s home become impossible, as did face-to-face conversation wherein important information could be identified and exchanged.

Another challenge of the allocation process was describing psychological needs: *“…To allocate services, we must describe how a service can help with anxiety, we are struggling to put it into words... Physical needs win the duel against those with ‘just’ anxiety, to say it a little simplistically... I ask myself why do we not manage to reach through with these psychological needs?”* The challenge of describing psychological needs could prevent these needs from being included in the assessment or from being allocated services for.

A lack of services to allocate further challenged the purchasers. When the purchasers described the different services available to meet psychosocial needs (e.g., forest-hiking groups or individual conversations for support), most were not available for PWDs; they were instead intended persons with good cognitive functioning. The services the purchasers felt able to allocate were mainly day care centres, technical aids such as forget-me-not calendars, or respite care and dialogue groups or educational programmes for relatives. The allocation of extra time to provide services to PWDs within the HCSs was uncommon. The day care centres become a “collective service” allocated to cover all individual needs.

How the purchasers viewed the different “groups” of service applicants could also influence the allocation. A purchaser described that both having dementia and being old could lead to disfavour when allocating services to meet psychological needs: *“…I think it has to do with age and that we think differently about PWDs… This is a backdrop to why we don’t allocate services to meet psychological needs. We allocate, for instance, individual conversations with psychiatric nurses to support younger persons; we also could have done the same for PWDs. However, younger people will participate more in society. It’s about our traditional way of thinking”.* Further, some purchasers perceived PWDs as receiving limited benefits from these types of services due to their impaired cognition. An adjustment in relation to age and cognitive capacity was undertaken.

Finally, several purchasers had the impression that the HCS providers had difficulties understanding the intention and scope underlying an administrative decision allocating a short visit to “only” look after a PWD. These purchasers perceived providers as having limited knowledge of the importance of meeting psychosocial needs. Some purchasers explained that they had the opportunity to allocate a short visit to prevent loneliness or anxiety but refrained from doing so owing to their view of the provider’s inability to implement the decision. One explained: *“If we allocate ‘only a visit’, then they are quick to say that the visit is unnecessary, we are not doing anything there, we just open the door say hello and leave… They do not see that this short visit may prevent unrest and anxiety. It’s about knowledge, limited time and attitudes”.* Some purchasers circumvent this problem by sneaking in a short visit to see how the PWD was doing, describing this service as a practical task in the administrative decision: *“…it’s about the attitude towards these types of visits, they have to do something concrete. Hence, we try to connect a visit to something practical… like seeing that the stove is turned off”.* A practice had developed where the allocation of HCSs was adjusted in relation to the purchasers’ perceptions of the providers’ attitudes towards delivering certain services.

### A challenging documentation of administrative decisions

The purchasers perceived formulating specific administrative decisions regarding psychosocial needs as challenging: *“…it is much easier to write and formulate an administrative decision that focuses on physical needs, it is more tangible and easier to describe… You also have to weigh your words not to offend; with regard to social and psychological needs, it’s slightly more taboo”.* Although the purchasers often described both social and psychological needs as challenging to formulate in administrative decisions, our analysis of the administrative decisions showed that social needs were described in all administrative decisions allocating day care centres (Table 3). An administrative decision allocating day care centre could be formulated as follows: *“You live alone and express that your days are long and lonely. You feel alone… In the day care centre, you will meet others in a socially organized environment…”.* In administrative decisions allocating HCSs, social needs were described to a smaller extent (Table 3). When they were described, it was often done in brief terms: *“Widow, living alone... a son lives nearby, helping with dinner”.*

Psychological aspects were seldom described in the administrative decisions (Table 3), although the purchasers perceived services to meet psychological needs as being especially important to PWDs. However, when it was done, the description was formulated in a relatively clear manner: *“You are often troubled with anxiety and unrest. It’s difficult for you to live in your own home, as you are scared of being alone…You are granted daily supervision from the HCSs to secure nutrition and to give you a feeling of security because of anxiety and problems with unrest…”.* However, such descriptions were uncommon; psychological aspects were rarely described beyond standardized formulations stating that the allocation of services was based on a comprehensive assessment: *“The administrative decision is based on a physical, social and psychological assessment…”.*

## Discussion

This study provides new insights into the process of allocating services to meet the psychosocial needs of older home-dwelling PWDs. The findings show that the purchasers’ desire to conduct a comprehensive allocation process is challenged. A web of different interplaying aspects, which the purchasers had to consider throughout the entire allocation process, influences their deliberations, showing the complexity of the allocation process, which is discussed below from two perspectives: deliberations influenced by two different hierarchies and a supply-led allocation process.

### Deliberations influenced by two different hierarchies

The first hierarchy was a hierarchy regarding needs. PWDs’ needs were rated into different levels of importance for assessment and of assessment difficulty. Physical needs were rated as the most important when assessing for HCSs, followed by social needs, and psychological needs were rated as the least important. That physical needs took precedence is supported by previous studies finding that older home-dwelling persons’ physical needs are emphasized and carefully assessed more than their psychosocial needs [20, 21]. However, these studies were not dementia-specific. That social, and especially, psychological needs are not emphasized to a larger degree during assessments for PWDs, as found in this study, is surprising, since PWDs have an increased risk of experiencing social isolation [13], and neuropsychiatric symptoms are significantly more common among PWDs than among persons without dementia receiving HCSs [28]. Psychosocial needs were not assessed at the same level as physical needs, underscoring the ambiguity and complexity of the psychosocial concept [22]. The purchasers’ division of the psychosocial concept into a social part and a psychological part might help the purchasers better understand and operationalize the concept [8]. However, this division also introduces a risk of omitting important dimensions. For example, cultural or spiritual needs were described in neither the focus group interviews nor the administrative decisions. The purchasers further rated the assessment and documentation of needs into different levels of difficulty, supporting the view that unclear and difficult concepts complicate the assessment. Our finding that psychological needs were perceived as the most challenging to assess and describe might be related to their intangibility and perceptual grounding, not explicit objective traits [48]. When needs that are perceived as the least important to assess are also perceived as the most challenging to assess and describe, these needs are at risk of being unassessed, irrespective of their importance to PWDs.

The second hierarchy rated different “groups” of applicants. In accordance with governing regulation, age alone should not be a disqualifying factor for receiving services [49]. Nevertheless, this study indicates that old age seemed to reduce applicants’ chances of receiving services to meet psychological needs within the HCSs. This finding is not unique. When resources are scarce, services for older recipients seem to lose out [50]. In a systematic review examining factors that influenced care managers’ resource allocation, Fraser and Estabrooks [34] found that impaired cognition often increased service allocation. This finding partly contradicts the results of this study, where impaired cognition was described as inhibiting the allocation of a range of services. In this study, having dementia and being old especially seemed to influence the allocation of services related to psychological needs. For example, individual conversations with psychiatric nurses could not be allocated because purchasers were uncertain whether PWDs benefit from these types of services, given their impaired cognition. Indeed, impaired cognition might influence PWDs’ ability to benefit from certain types of services. However, it is surprising that impaired cognition could act as a disqualifying factor for receiving services, since impaired cognition alone might increase PWDs’ need for assistance to address symptoms such as depression, anxiety, and unrest. In a hierarchy rating different groups of applicants, older PWDs seemed to be exposed to a double disqualification, being old and having dementia.

### A supply-led allocation process

This study showed that the allocation process was more supply-led than need-led. Similar findings have previously been found in studies examining the assessment and allocation of community care to older persons in general [22, 25]. Despite an expressed wish to allocate services based on the individual needs of the PWD, a lack of services to allocate greatly influenced the allocation. Limited available services also influenced the extent to which different needs were assessed. One might question this incongruity when the purchasers state the importance of conducting a comprehensive assessment while simultaneously describing a practice where significant needs are omitted from the assessment. This incongruity might be understood by considering the limited available services to allocate, especially for psychological needs. Even if psychological needs were assessed and identified, a lack of services adversely affected the allocation [24]. Against this backdrop, it might be deemed appropriate to utilize the limited time available to assess only those needs that can be met by available services. In contrast, if the assessments are mainly based on available services, then the prospect for many PWDs to have their psychological needs met seems scant.

The complexity within the allocation process went beyond definitional challenges, a lack of time, and limited services to allocate. A surprising finding was that services for meeting psychosocial needs were often not allocated within the HCSs due to the purchasers’ perception of the providers as reluctant to provide these services, or lacking knowledge to do so. This finding indicates that, in cases where the purchasers could have allocated, for instance, a short visit to prevent unrest, services were not allocated. However, some purchasers described circumventing this reluctance by allocating a practical task (e.g., seeing if the stove is turned off) as a response to psychosocial needs. In this manner, the reason for the service was consciously camouflaged as a practical task. One might question whether this practice responds to the actual needs of PWDs. Indeed, providers’ knowledge and understanding of how to meet psychosocial needs may vary [51, 52]. However, by not describing services to meet such needs within the administrative decisions, the purchasers might have unconsciously contributed to reducing the fulfilment of psychosocial needs. Not describing psychosocial needs in the administrative decisions might decrease the possibility that HCS providers will meet these needs, especially when services are provided in strict accordance with what is stated in the administrative decisions [53]. That 142 of the 184 administrative decisions allocating HCSs described neither social nor psychological aspects might indicate that the purchasers’ viewed the fulfilment of physical needs as the main task of the HCSs [54]. A desire to consider the already “overloaded” HCS providers might similarly influence allocation. It is interesting to see that the purchasers admitted to being influenced by organizational issues and not using their discretion to a greater degree [24]. Evidently, the purchasers felt obliged to act based on available services, at the expense of the PWDs’ needs and their own professional standards. There was a mismatch between the purchasers’ ideal of conducting a comprehensive need-led allocation process and their actual practice [55]. However, the purchasers found themselves in a challenging situation, trying to balance the contradiction between being PWD advocates while simultaneously acting as organizational gatekeepers [33]. The purchasers’ loyalty to the organization appeared to be greater than their loyalty to PWDs [22, 24]. Therefor a shift is needed from emphasizing organizational aspects within the allocation process to a more relational focus emphasizing the needs of PWDs [21]. Such a shift is of great significance for meeting PWDs’ psychosocial needs within HCSs.

### Limitations

All of the participants were women. This study might have benefited from having male participants. However, the sample is representative of the service, which is mainly staffed by women. Another limitation that may restrict the transferability of this study to other contexts is the difference in how various countries have structured their HCSs. Nonetheless, this study has identified challenges during the allocation process that may have relevance independently of service organization.

### Further research

More research is needed to explore the allocation process further, including aspects influencing the purchasers when they assess and allocate services to meet the psychosocial needs of older home-dwelling PWDs. More knowledge is needed about the best-practice requirements that would enable healthcare professionals to adequately define and describe psychosocial needs, to ensure a comprehensive allocation process. Studies emphasizing a more critical perspective towards the allocation process itself and its use in assessing and allocating healthcare services to older home-dwelling PWDs should be encouraged. Further research should also address how PWDs and their relatives experience the consideration of their psychosocial needs within the allocation process.

## Conclusions

This study revealed a complex allocation process. The purchasers viewed a comprehensive allocation process as significant. However, a web of different interplaying aspects that the purchasers had to consider prevented them from conducting a comprehensive allocation process based on the needs of PWDs. A feeling of obligation to conduct a supply-led allocation process became apparent, at the expense of a more comprehensive, need-led approach. Conducting a comprehensive allocation process is important, especially for PWDs, since these persons often have challenges safeguarding their own psychosocial needs. A dyadic focus that better balances organizational issues and the needs of PWDs is of great importance for meeting PWDs’ psychosocial needs. Psychological needs were especially prone to remaining unassessed, and in addition the associated services available to allocate were lacking. Increased knowledge to better capture and describe these blurry and sensitive needs is important to preventing these needs from remaining unassessed and unfulfilled. Not assessing or describing psychological needs might obscure the need for services, resulting in the non-implementation of additional or new services. Having varied and sufficient services to allocate is important, but not sufficient. Psychosocial needs must be better incorporated as a significant element throughout the entire allocation process. Increasing knowledge of aspects that influence the allocation process contributes to developing an allocation process that is better adapted to capture the psychosocial needs of older home-dwelling PWDs.

## Additional file

Additional file 1: Interview guide. Manuscript: Purchasers’ deliberations on psychosocial needs within the process of allocating healthcare services for older home-dwelling persons with dementia: A qualitative study. (DOCX 14 kb)

## Abbreviations

HCS(s): Home care service(s)
PWD(s): Person(s) with dementia
REC: Regional Committee for Medical and Health Research Ethics
