# Supplementary material for: Purchasers’ deliberations on psychosocial needs within the process of allocating healthcare services for older home-dwelling persons with dementia: a qualitative study
Source: BMC Health Serv Res. 2018 Oct 1;18:746. doi: 10.1186/s12913-018-3550-7 (PMC6167900; doi:10.1186/s12913-018-3550-7)
Supplement: Supplementary file 1 — Interview guide. Manuscript: Purchasers’ deliberations on psychosocial needs within the process of allocating healthcare services for older home-dwelling persons with dementia: A qualitative study. (DOCX 14 kb) [file 12913_2018_3550_MOESM1_ESM.docx]

**Additional file 1.**

Manuscript: Purchasers’ deliberations on psychosocial needs within the process of allocating healthcare services for older home-dwelling persons with dementia: A qualitative study

**Interview guide**

- We have two of your administrative decisions here, one regarding home care services and one regarding day care centres. Can you please comment on these administrative decisions and explain in what way psychosocial health and needs are made visible?
- Can you please describe how and what you consider and emphasise when you are in PWDs’ homes assessing their needs? (e.g., how are you thinking? What thoughts and reasoning processes lie behind the assessment? How is your decision made? What is the setting for the charting conversation?)
- What do you think “psychosocial health” implies?
- If we look more closely at psychosocial health, what do you do to identify related needs? (e.g., assessment tools? Observations? Network mapping? What kinds of questions are asked? Who’s assessment of PWDs’ needs emphasises are emphasised the most - PWD/relatives?)
- In your experience, what psychosocial needs do PWDs have? (e.g., PWDs’ own perception/wishes, relatives, municipalities? Best suited service? Most common allocated service?)
- In what way are psychosocial needs described in the administrative decisions?
- To what extent would you say that psychosocial needs have been addressed in the administrative decisions?
- Do you experience having sufficient focus on patients' psychosocial health? ( e.g., Why/why not? Physical/psychosocial needs - differences?)
- Are meeting psychosocial needs part of necessary healthcare that shall be provided by the municipalities or is it not to be regarded as necessary healthcare? Why?
- If psychosocial health should be attended optimally, how should the administrative decisions be?
